# The Protective Effects of 2,3,5,4′-Tetrahydroxystilbene-2-*O*-β-d-Glucoside in the OVA-Induced Asthma Mice Model

**DOI:** 10.3390/ijms19124013

**Published:** 2018-12-12

**Authors:** Yun-Ho Hwang, Su-Jin Kim, Hangun Kim, Sung-Tae Yee

**Affiliations:** 1 College of Pharmacy, Sunchon National University, 255 Jungangno, Suncheon 540-950, Korea; hyh7733@naver.com (Y.-H.H.); ksz1353@naver.com (S.-J.K.); hangunkim@sunchon.ac.kr (H.K.); 2 Suncheon Research Center for Natural Medicines, Suncheon 540-950, Korea

**Keywords:** 2,3,5,4′-tetrahydroxystilbene-2-O-β-d-glucoside (TSG), asthma, airway hyperresponsiveness (AHR), inflammation

## Abstract

Asthma is an inflammatory disease caused by an imbalance of Th1 and Th2 cells. In general, asthma is characterized by a stronger Th2 response. Most conventional asthma treatment focuses on improving airway flow or suppression of airway inflammation. To reduce the side effects of currently used asthma medicines, we have conducted studies on natural products that have no side effects. 2,3,5,4′-tetrahydroxystilbene-2-O-β-d-glucoside (TSG), the main compound of *Polygonum multiflorum* (PM), has various biological activities, including anti-inflammation and anti-oxidation activities. However, the effect of TSG on asthma has not been studied yet. We examined the effects of TSG on Th2 immune responses using an OVA-induced asthma animal model. OVA-sensitized mice were treated with TSG. 24 h after the last intranasal challenge, airway hyperresponsiveness (AHR) was measured or serum and bronchoalveolar lavage fluid (BALF) were harvested. We measured typical Th1 and Th2 cytokines in serum and BALF. As a result, TSG suppressed Th2 responses, as shown by the lower levels of IL-4, IL-5, total IgE, OVA-specific IgE, and OVA-specific IgG1. On the other hand, TSG increased Th1 responses, as shown by the levels of IFN-gamma. Collectively, these results confirm the potential of TSG for asthma treatment through modulation of inflammatory responses. Considering that the cytotoxic effect of PM extract is due to the cis isomer of TSG, if the effect of TSG on asthma treatment is found to be non-toxic in clinical trials, it would be more effective to use it as a purified component than PM extract as an asthma treatment agent.

## 1. Introduction

Asthma is an inflammatory disease caused by an allergic reaction. Symptoms include wheezing, coughing, chest tightness, and dyspnea due to airway obstruction [1]. According to the World Health Organization (WHO), asthma affects 300 million people worldwide and about 250,000 people die each year [2,3]. Asthma occurs in all ages, including children, adults and the elderly, and the incidence of asthma is increasing in many countries [4,5,6,7]. Therefore, an increase in the incidence of asthma leads to an increase in the cost of treatment, which puts a burden on individuals and countries [2].

Approximately 95% of asthmatic patients respond effectively to corticosteroids and β2-agonists, and their proper use does not adversely affect them. However, despite this treatment accounting for 50% of asthma health care costs, 5–10% of patients do not respond well to this therapy [8]. Moreover, corticosteroids are recognized as the first line of treatment for adult asthma but are not widely accepted in children with asthma due to side effects such as growth inhibition in children with asthma [9]. Consequently, efforts should be made to find new anti-asthma treatments that effectively alleviate asthma symptoms [10].

Medicinal plants are plants used for therapeutic purposes and for the isolation of useful drugs. Herbs can be divided into two major parts: The complex of the mixture includes a wide variety of compounds, and the second category refers to pure, chemically-defined active principles. *Polygonum multiflorum Thunb.* (PM) is well known as He Shou Wu in China and Fo-Ti in North America. Pharmacological studies have emphasized the benefits of PM in the treatment of a variety of diseases, such as cancer, diabetes, alopecia, atherosclerosis, and neurodegenerative diseases [11]. 2,3,5,4′-tetrahydroxystilbene-2-O-β-d-glucoside (TSG) is known as a major active single compound of PM [12], and presents a variety of biological effects, such as anti-osteoporosis [13], anti-inflammation [14], kidney protection [15], anti-cancer [16], and liver protection effects [17]. However, Lei *et al.* reported that PM extracts have hepatotoxicity [18], and this is probably due to the cis isomer of TSG [19]. This suggests that the use of PM extracts for the treatment of asthma poses a risk of hepatotoxicity, even if TSG is the main compound of PM extract. Although many biological activities of TSG have been reported, the effect of TSG on asthma has not been reported yet.

In this study, we demonstrate the effects of TSG on airway hyperreactivity and Th2 responses in mice sensitized and challenged with OVA. We sensitized mice against OVA and challenged them with OVA at regular intervals. We administered TSG to OVA-induced asthmatic mice and measured Th1/Th2-mediated immune responses. The results of this study demonstrate that the injection of TSG into asthmatic mice alleviates asthma symptoms by inhibiting the Th2 immune response.

## 2. Results

### 2.1. Effects of TSG on Airway Hyperresponsiveness (AHR)

**Figure 1:**
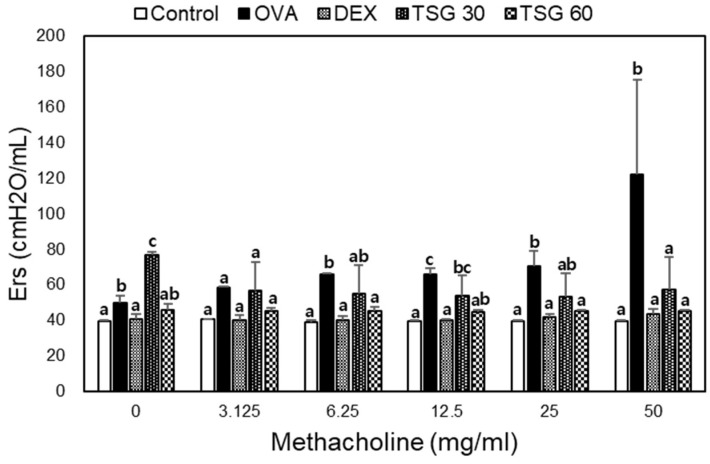
Assessment of allergen-induced airway hyperresponsiveness by the forced oscillation technique. Elastance (Ers) was determined in OVA-sensitized mice challenged with OVA after IP infection of 2,3,5,4′-tetrahydroxystilbene-2-O-β-d-glucoside (TSG) at two concentrations (groups TSG30 and TSG50), dexamethasone (DEX), or saline (OVA); control mice were challenged with saline alone. a, b, and c: The means not sharing a common letter are significantly different between groups at *p* < 0.05 as measured by one-way ANOVA with Duncan’s multiple-range test.

In order to assess the effects of 2,3,5,4′-tetrahydroxystilbene-2-O-β-d-glucoside (TSG) on their airway functions, the OVA or TSG treated mice were exposed to methacholine (MCh), and AHR was measured through the FlexiVent. We compared the development of allergic asthma in the presence or absence of TSG in the OVA asthma murine model, as depicted in Figure 1. At a concentration of 3.125 to 50 mg/mL of methacholine, the elastance (Ers) in the OVA group was significantly increased compared to the control group. The treatment of dexamethasone (Dex) suppressed AHR response. Furthermore, treatment of OVA-sensitized mice with TSG before challenge decreased the Ers. This result suggests that administration of TSG inhibits AHR, which is one of the features of asthma.

### 2.2. Effects of TSG on the Inflammatory Cell Population in the BAFL and Blood

**Figure 2:**
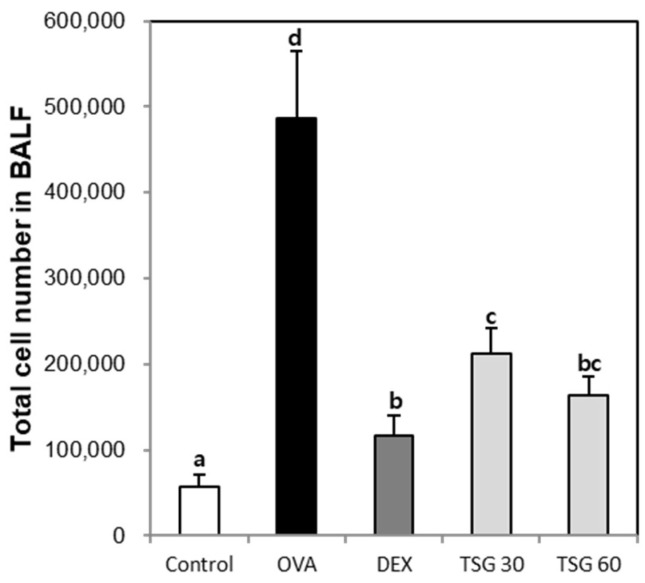
The effect of TSG on total cell number in the bronchoalveolar lavage fluid (BALF). Mice were sacrificed 24 h after the last challenge, and the lungs were lavaged four times with 0.5mL ice-cold saline via a tracheal cannula. The BALF was then centrifuged and the pellets were re-suspended with 1000 μL and used for cell counting. Group data are expressed as means ± SD for five mice per group. a, b, c, and d: The means not sharing a common letter are significantly different between groups at *p* < 0.05 as measured by one-way ANOVA with Duncan’s multiple-range test.

**Figure 3:**
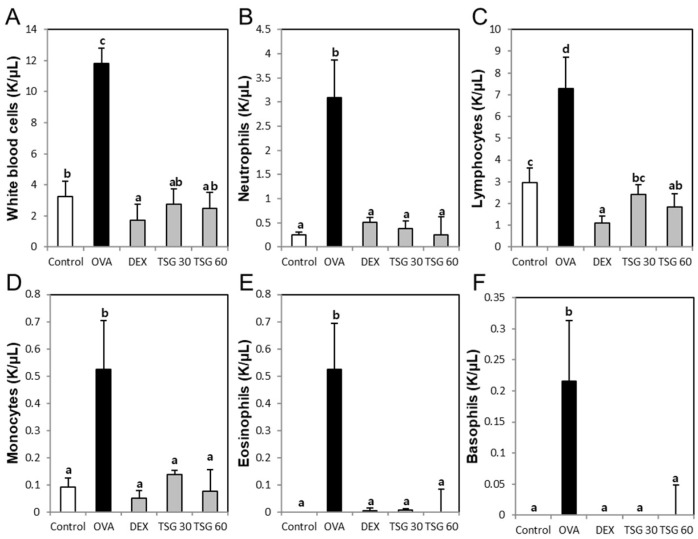
The effects of TSG on the population of inflammatory cells in the blood. (**A**) white blood cell, (**B**) neutrophil, (**C**) lymphocyte, (**D**) monocyte, (**E**) eosinophil, and (**F**) basophil were analyzed by the HEMAVET 950. Group data are expressed as means ± SD for five mice per group. a, b, c, and d: The means not sharing a common letter are significantly different between groups at *p* < 0.05 as measured by one-way ANOVA with Duncan’s multiple-range test.

In order to determine the effect of TSG on the changes of inflammatory cell populations, we measured changes in inflammatory cells using an animal blood cell analyzer (HEMAVET950). The bronchoalveolar lavage fluid (BALF) total cell number in the OVA group was increased compared to the control group. The enhancement of the BALF total cell number in the OVA group was decreased by exposure of TSG 30 and 60 mg/kg (Figure 2). Also, white blood cells (Figure 3A), neutrophils (Figure 3B), lymphocytes (Figure 3C), monocytes (Figure 3D), eosinophils (Figure 3E), and basophils (Figure 3F) in the OVA asthmatic group were significantly higher than in the control group. However, this upregulation of the inflammatory cell population in the OVA group was reversed by the treatment with TSG. These results indicated that TSG has an inhibitory effect on the influx of inflammatory cells in the nasal mucosa.

### 2.3. Effects of TSG on the Production of Cytokines in OVA-Induced Asthmatic Mice

**Figure 4:**
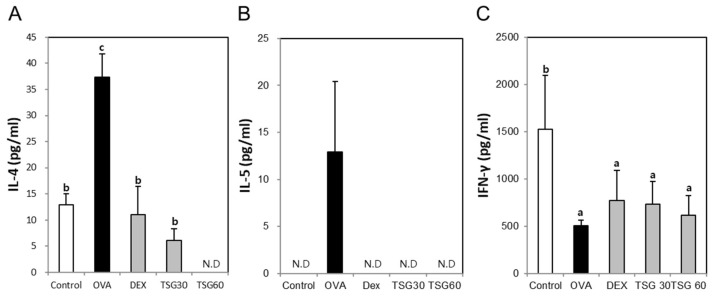
The effects of TSG on Th1-mediated cytokine and Th2-mediated cytokine in BALF. (**A**) IL-4, (**B**) IL-5, and (**C**) IFN-gamma were measured by enzyme-linked immunosorbent assay (ELISA). Group data are expressed as means ± SD for five mice per group. a, b, and c: The means not sharing a common letter are significantly different between groups at *p* < 0.05 as measured by one-way ANOVA with Duncan’s multiple-range test.

To determine the effect of TSG on Th1 or Th2 cytokine changes, we measured IL-4, IL-5, and IFN-γ. The BALF IL-4 and IL-5 in the OVA group were increased in comparison with that of the control. However, the exposure of DEX in the asthmatic mice decreased the production of the IL-4 and inhibited IL-5 production. The IL-4 in the TSG 30 mg/kg group was significantly decreased in comparison with that of the OVA group. The production of IL-4 in the TSG 60 mg/kg was not detected (Figure 4A). Also, the production of IL-5 was not detectable after TSG treatment (Figure 4B). On the other hand, the production of IFN-γ in the OVA group was lower than in the control group. The treatment of DEX, TSG 30, and TSG 60 mg/kg were lower than in the control group (Figure 4C). However, it was not statistically significant. These results indicated that TSG suppressed the IL-4 or IL-5 and increased the production of IFN-γ.

### 2.4. Effects of TSG on the Release of Immunoglobulin Class Switching in Serum

**Figure 5:**
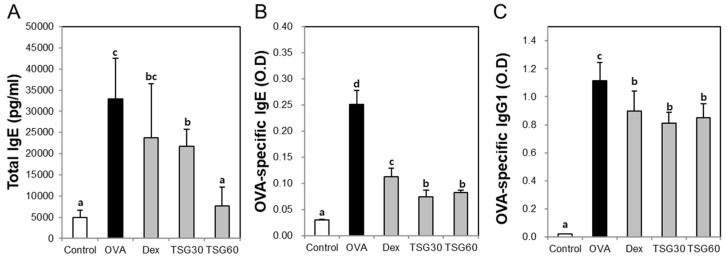
The effects of TSG on Th1 and Th2-mediated immunoglobulins (IgG’s) in serum. (**A**) total IgE, (**B**) OVA-specific IgE, and (**C**) OVA-specific IgG1 were measured by ELISA. Group data are expressed as means ± SD for five mice per group. a, b, c, and d: The means not sharing a common letter are significantly different between groups at *p* < 0.05 as measured by one-way ANOVA with Duncan’s multiple-range test.

To confirm the production of immunoglobulin associated with Th2 cytokines, we measured total IgE, OVA-specific IgE, and OVA-specific IgG1 from the serum. The levels of total IgE and OVA-specific IgE in the OVA group were significantly increased in comparison with the control group. The treatment of the TSG in the OVA group significantly reduced the level of total IgE and OVA-specific IgE (Figure 5A,B). Furthermore, OVA-specific IgG1 in the OVA group was higher than the control group. The treatment of DEX and TSG (30 and 60mg/kg) significantly inhibits the level of OVA-specific IgG1 (Figure 5C). These results suggest that TSG suppresses the levels of the IgG’s (IgE and IgG1) associated with the Th2 response.

### 2.5. Effects of TSG on Histological Changes in Asthmatic Mice

**Figure 6:**
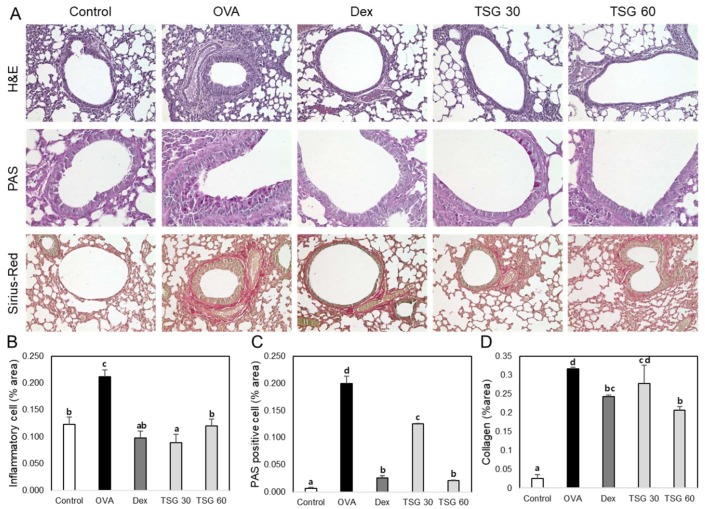
The effect of TSG on the histology of lung tissue in the OVA-induced murine model of asthma. The C57BL/6 mice were sensitized and challenged with OVA for asthma induction. At the end of the experiment, the mice lungs were removed. (**A**) The lungs were stained by H&E (×200), PAS (×400), and Picro Sirius Red (×200). The percentages of (**B**) inflammatory cells, (**C**) PAS-positive cells, and (**D**) collagen in the lung sections were measured via Image J program. The data represent three mice per group. All data were expressed as means ± SD. a, b, c, and d: The means not sharing a common letter are significantly different between groups at *p* < 0.05 as measured by one-way ANOVA with Duncan’s multiple-range test.

**Figure 7:**
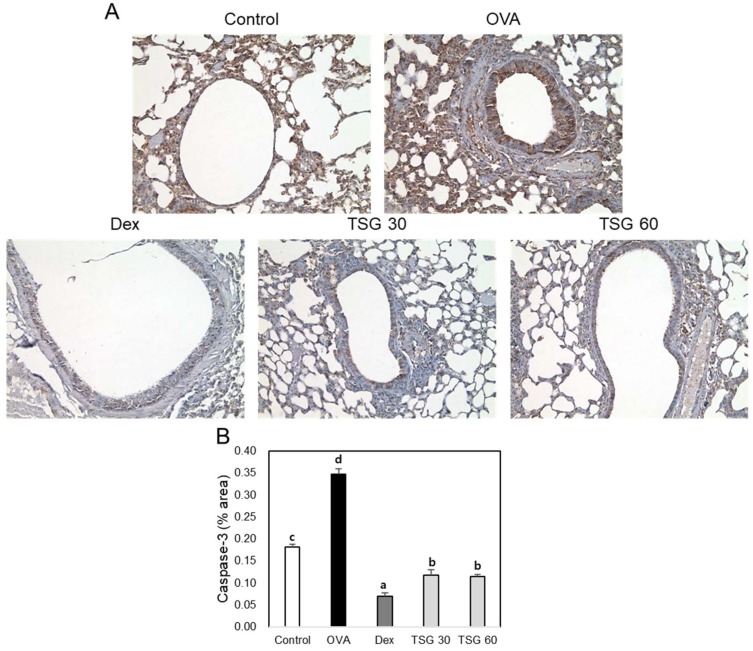
The effect of TSG on immunohistochemistry in the lung tissues of the OVA-induced murine model of asthma. C57BL/6 mice were sensitized and challenged with OVA for asthma induction. At the end of the experiment, the mice lungs were removed. (**A**) The lungs were stained by caspase-3 (×200) immunohistochemistry. The percentages of (**B**) caspase 3-positive cells in the lung sections were measured via Image J program. All data were expressed as means ± SD. a, b, c, and d: The means not sharing a common letter are significantly different between groups at *p* < 0.05 as measured by one-way ANOVA with Duncan’s multiple-range test.

In order to examine the effect of TSG on the lung tissues of OVA-induced asthma mice, we performed tissue stainings, such as hematoxylin and eosin (H&E), Periodic acid–Schiff (PAS), and Picro Sirius Red Stain (Figure 6A). The accumulation of inflammatory cells and the thickness of epithelial cells in the lung tissues of the OVA group were higher than those of the control group. However, the administration of TSG to the OVA group significantly reduced the accumulation of inflammatory cells (Figure 6B). Mucus-producing goblet cells in the lungs were quantified using PAS staining. As a result, mucus was produced more in the asthma animal model than in the control group. Increased mucus was significantly reduced by TSG administration (Figure 6C). Collagen deposition was increased in the lungs of the OVA group than in the control group, which was reduced by administration of TSG (Figure 6D). Furthermore, we observed an increase in the expression of caspase-3 in the lungs of OVA-induced asthmatic mice. However, exposure of TSG inhibited the increased expression of caspase-3 (Figure 7). These results indicated that the TSG has a suppressive effect on lung inflammation, mucus production, and lung fibrosis.

## 3. Discussion

Asthma medications can be classified as controllers or relievers. Controllers are medications taken daily on a long-term basis for clinical control through anti-inflammatory effects. Relievers act quickly to reverse bronchoconstriction and relieve symptoms. However, these therapies cause a variety of side effects [20]. Bupleurum, Cordyceps, Ephedra (Ma huang), Ginkgo, Licorice, Magnolia, Pinellia, Platycodon, Polygonum, and Scute have been used for the treatment of asthma [21,22]. However, many cases of liver damage associated with PM have been reported worldwide; PM has liver toxicity and may cause different degrees of liver damage. Long-term or high-dose administration of PM may cause various symptoms, such as fatigue, anorexia, nausea, yellowing of skin and sclera, and yellow urine [18]. 2,3,5,4′-tetrahydroxy trans-stilbene-2-O-β-d-glucoside (trans-SG) and its cis-isomer were the two major compounds in EA extract of PM, and higher contents of cis-SG were detected in PM liquor or preparations from actual liver intoxication patients associated with PM compared with generally collected samples [19]. As previously stated, Polygonum has been used for the treatment of asthma, but long-term or high-dose use of it can cause a variety of side effects, including hepatotoxicity, due to the presence of the cis isomer of TSG. From this viewpoint, asthma treatment using PM extract has the potential to cause various side effects. Nevertheless, PM has various biological effects [13,14,15,16] and 2,3,5,4′-tetrahydroxystilbene-2-O-β-d-glucoside (TSG) as a main single compound has anti-inflammatory effects [14] and anti-osteoporosis effect [13]. In the present study, we investigated the effects of TSG on asthma as one of the inflammatory diseases.

Th1 cells mainly secrete IL-2 and IFN-γ, which causes a cellular immune response. On the other hand, Th2 cells secrete IL-4, IL-5, and IL-13, and promote humoral immune responses [23]. IL-4 induces the migration of leukocytes to the site of inflammation [24]. IL-5 is essential for maturation of eosinophil in the bone marrow [25]. Accumulation of eosinophils induces airway hyperresponsiveness (AHR) and airway remodeling [26]. Accumulating evidence has shown that altering the cytokine production of Th2 cells by inducing Th1 responses prevents Th2-related diseases such as asthma and allergies. The elimination of allergies is related to the normalization of the IFN-γ. Since IFN-γ exerts a direct inhibitory effect on Th2 cytokines and reduces IL-4 and IL-5 production levels, IFN-γ secreted from Th1 cells inhibits the Th2 response leading to allergic disease [27]. In the present study, administration of TSG to OVA-induced asthmatic mice decreased the level of Th2-associated cytokines (IL-4 and IL-5) in the BALF and inflammatory cells such as eosinophils, basophils, neutrophils, monocytes, lymphocytes. Moreover, TSG inhibits the levels of total IgE, OVA-specific IgE, and OVA-specific IgG1. These results indicated that the reduction of antigen (OVA)-specific IgE and IgG1 shows a decrease in IL-4, demonstrating that TSG reduces the Th2 immune response. On the other hand, the level of IFN-r as a Th1-associated cytokine is increased by administration of TSG. These results indicated that TSG inhibits the Th2-associated cytokines such as IL-4 and IL-5 and increased IFN-r as a Th1-associated cytokine.

The anti-inflammatory drug dexamethasone used as a positive control in this study is known to inhibit the production of inflammatory cytokines, the accumulation of chronic inflammatory cells including eosinophils and T lymphocytes, and the development of subepithelial fibrosis and epithelial hypertrophy [28]. In the asthmatic mice induced by sensitization and provocation of airway inflammation with OVA, production of IL-4 and IL-5 in the BALF and inflammatory cells such as leukocytes, neutrophils, lymphocytes, monocytes, eosinophils, and basophils were increased, which enhanced AHR. However, intraperitoneal administration of TSG (30 and 60 mg/kg) reduced these inflammatory responses similarly to dexamethasone. The TSG concentrations used in this study were also applied in other studies. Some research showed that TSG pretreatment at the doses of 30 and 60 mg/kg not only inhibited the production of pro-inflammatory cytokines induced by LPS, such as IL-1β, IL-6, and tumor necrosis factor-α, but also prevented the LPS-induced enhancement of oxido-nitrosative stress in the mouse hippocampus and prefrontal cortex [29]. The TSG used in this study may be effective in treating inflammatory diseases. Moreover, in the animal experiment, no adverse events such as convulsions, dyspnea, or death were observed during administration of TSG. At autopsy, no abnormal changes were observed in major organs such as the liver, heart, kidney, thymus, and spleen. However, in this study, acute and chronic toxicity assessment of other organs including hepatotoxicity was not performed, and safety was not investigated. This represents a limitation for this experiment. Therefore, a safety assessment of the drug must be performed before TSG can be used in patients with asthma. In a previous study, TSG (100, 200, 400, 800 mg/kg) alone had no effect on serum ALT/AST activity in mice. Also, results of liver histological evaluations showed that no obvious pathological changes occurred in mice treated with TSG (800 mg/kg) alone [30]. 

Many studies using OVA-induced asthma animal models have been conducted to determine the therapeutic effects of natural compounds on asthma. The natural compounds such as baicalin [31], forsythiaside A [32], polydatin [33], aloperine [34], and curcumin [35] have been reported to be effective in the prevention and treatment of asthma in asthma models using OVA. Although TSG does not have a better effect than these natural compounds, we think that TSG has effects similar to natural compounds or dexamethasone. Side effects of corticosteroids can occur in both children and adults and are reported to have side effects on the central nervous system, cardiovascular, gastrointestinal, kidney, and musculoskeletal systems [36]. TSG is the main compound of PM extract. The use of PM extracts for the treatment of asthma may be dangerous due to hepatotoxicity caused by the cis isomer of TSG. If TSG is not toxic in clinical trials, purified TSG will be more effective in treating asthma than PM extracts.

## 4. Materials and Methods

### 4.1. Reagents

2,3,5,4′-tetrahydroxystilbene-2-O-β-d-glucoside (TSG, CAS Number: 82373-94-2, purity: ≥98%) and ovalbumin (A5503) were purchased from Sigma-Aldrich (Louis, USA). To measure inflammatory cytokines in serum and bronchoalveolar lavage fluid (BALF), we purchased purified rat anti-mouse IL-4, purified rat anti-mouse IL-5, purified rat anti-mouse IFN-γ, biotin rat anti-mouse IL-4, biotin rat anti-mouse IL-5, biotin rat anti-mouse IFN-γ, purified rat anti-mouse IgE (R35-72), purified rat anti-mouse IgG1 (A85-3), biotin rat anti-mouse IgE (R35-118), biotin rat anti-mouse IgG1 (A85-1), and biotin rat anti-mouse IgG2a (19-5) from BD Biosciences (San Diego, CA, USA).

### 4.2. Animals

Eight-week-old female C57BL/6 mice were bred and maintained under specific pathogen-free conditions at ORIENT BIO (Seongnam, Korea). Animals were housed in standard polycarbonate cages under controlled conditions (22 ± 2 °C, RH 50%–60 %, and a 12-hlight/dark cycle). The mice were housed in polycarbonate cages and fed a standard animal diet with water. In order to minimize the pain caused by the surgical procedure, animal experiments were performed under zoletil/rompun anesthesia. All mice were treated in strict accordance with the Sunchon National University Institutional Animal Care and Use Committee’s (SCNU IACUC) guidelines for the care and use of laboratory animals. All procedures were approved by the SCNU IACUC (permit number: SCNU IACUC-2018-08).

### 4.3. Sensitization and Provocation of Airway Inflammation with OVA

**Figure 8:**
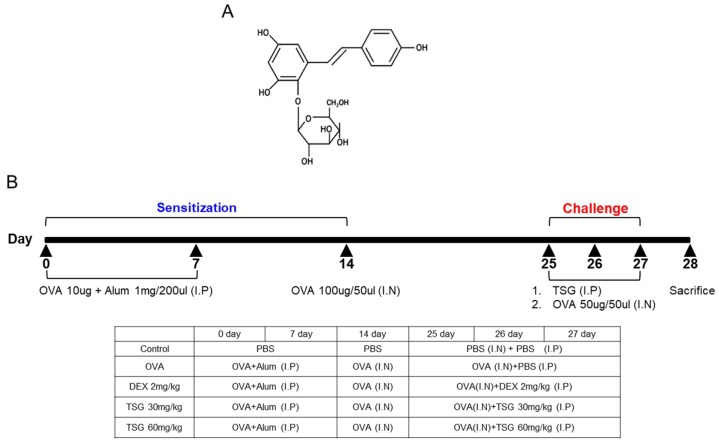
(**A**) Structure of 2,3,5,4′-tetrahydroxystilbene-2-O-β-d-glucoside (TSG) and (**B**) experimental protocol for induction of airway inflammation along with treatment scheme.

The mice were randomly divided into five groups (*n* = 5): The control group, OVA group, and TSG (at doses of 30 and 60 mg/kg, respectively) + OVA groups. The C57BL/6 mice were sensitized by the intraperitoneal injection of 10 μg of OVA and 1 mg of Imject Alum (Pierce) in 0.2 mL of saline on days 0 and 7. On day 14, the mice were anesthetized with the inhalation by intranasal instillation of 100 μg OVA in 50 μL phosphate-buffered saline (PBS) or PBS alone for the negative control. On days 25, 26 and 27, the mice were again anesthetized and challenged by the intranasal instillation of 50 μg OVA in 50 μL PBS or PBS alone for the negative control. One hour before the challenge, TSG dissolved in sterilized water was diluted in PBS and injected intraperitoneally with 200 μL (30 mg/kg or 60 mg/kg TSG). As a positive control, OVA-sensitized mice were intraperitoneally injected with dexamethasone (DEX) at a concentration of 2 mg/kg (DEX 2 mg/kg, 200 uL, i.p.) instead of TSG (Figure 8). 24 h after the last intranasal challenge, blood was collected from the retro-orbital plexus. After centrifugation at 5000 rpm, 4 °C, 5 min, the serum was stored at −20 °C until assayed for immunoglobulins such as total IgE, OVA-specific IgE, and OVA-specific IgG1 by ELISA. After sacrificing mice through cervical dislocation, bronchoalveolar lavage fluid (BALF) of the mice was performed four times each with 0.5 mL of saline. After BALF is centrifuged, the supernatant of BALF obtained from 2 mL of instilled saline was stored at −20 °C until assayed for cytokines such as IL-4, IL5, and IFN-γ by ELISA. The red blood cells in BAL were removed by Tris-buffered ammonium chloride. The BALF total cells were counted using a hemocytometer. Also, inflammatory cells such as white blood cells, neutrophil, lymphocytes, monocytes, eosinophil, and basophil were measured in blood using a HEMAVET 950 (Drew Scientific, Inc., Oxford, UK).

### 4.4. Assessment of Airway Hyperresponsiveness on the TSG in OVA-Induced Asthmatic Mice

As performed in previous studies, we measured the respiratory system elastance (Ers) as the airway hyperresponsiveness (AHR) factor in OVA-induced asthmatic mice in the same way. AHR factors were determined before each challenge and after each dose of MCh [37]. Briefly, the final challenged mice by the OVA or TSG were anesthetized using a mixture of Zoletil and Rompun and the anesthetized mice were tracheostomized using an 18G metal cannula. The mice were then placed in a flow-type body plethysmograph and connected by the endotracheal cannula to a small-animal ventilator (FlexiVent, SCIREQ Inc., Montreal, QC, Canada). Doses of methacholine (MCh) were administered using a nebulizer (Aeroneb) and progressively doubled concentrations ranging from 0 to 50 mg/mL.

### 4.5. Measurement of Inflammatory Cytokine and Immunoglobulin Production in OVA or TSG Exposed Mice

The levels of the cytokines (IL-4, IL-5, and IFN-γ) in the BALF and immunoglobulins (the total IgE, OVA-specific IgE, and OVA-specific IgG1) in the serum were measured by enzyme-linked immunosorbent assay (ELISA). The lower detection limits of these assays were 1.11 pg/mL (IL-4, IL-5, and total IgE) and 10 pg/mL (IFN-γ). To measure the concentration of the OVA-specific IgE and OVA-specific IgG1 in the serum, we used the OVA as the capture Ag and biotinylated anti-mouse IgE or IgG1 mAb as the detection Ab.

### 4.6. Histological Analysis of Lung Tissue in OVA or TSG Exposed Mice

The left lungs of the mice were removed, transferred into 4% formalin for 24 h (room temperature) and subsequently transferred into PBS. The left lung was dehydrated using ethanol and xylene, embedded in paraffin, and 4μm sections were obtained. The paraffin-embedded lung sections were stained with hematoxylin and eosin (H&E), periodic acid-schiff (PAS), and pico-sirius red. Images of the lung tissue sections stained with H&E and PAS were acquired with a microscope equipped with a ×20 or ×40 objective lens. For immunohistochemistry, the paraffin-embedded sections were deparaffinized. The slides were washed at room temperature and hydrated. The endogenous peroxidase activity was then quenched with 3% hydrogen peroxidase. The sections were then blocked and the endogenous avidin and biotin were blocked, following the manufacturer’s instructions. Inflammatory cells/epithelium, PAS-positive cells, lung fibrosis, and the expression of caspase were analyzed by the Image J program [38].

### 4.7. Statistical Analysis

The data are presented as the means ± standard deviations (SDs). Statistically significant differences between groups were identified by one-way analysis of variance (ANOVA) using SPSS version 22 (Chicago, IL, USA) with Duncan’s multiple range test. In addition, *p* < 0.05 was considered to indicate statistical significance.

## 5. Conclusions

Generally, asthma is caused by an imbalance of Th1 and Th2 cells. The administration of TSG to OVA-induced asthmatic mice decreased the production of IL-4 and IL-5, which led to the reduction of inflammatory cells such as eosinophils, neutrophils, basophils, and white blood cells. In addition, TSG effectively inhibited AHR by decreasing inflammatory cell accumulation and mucus hypersecretion in the lungs. On the other hand, IFN-r as a representative cytokine secreted from Th1 cells was increased by treatment of TSG. From a therapeutic point of view, TSG is considered a good candidate for the treatment of asthma caused by the Th2 immune response.
